# Morphometric and molecular characterization of an unpigmented haemosporidian parasite in the Neotropical turnip-tailed gecko (*Thecadactylus rapicauda*)

**DOI:** 10.1017/S0031182022001421

**Published:** 2023-03

**Authors:** Nubia E. Matta, Leydy P. González, Mario Vargas-Ramírez, Gediminas Valkiūnas, Ananías A. Escalante, M. Andreína Pacheco

**Affiliations:** 1 Facultad de Ciencias, Departamento de Biología, Universidad Nacional de Colombia, Sede Bogotá, Carrera 30, No. 45-03, Bogotá 111321, Colombia; 2 Facultad de Ciencias, Estación de Biología Tropical Roberto Franco (EBTRF), Universidad Nacional de Colombia, Carrera 33 #33 −76, Villavicencio 500005, Meta, Colombia; 3 Instituto de Genética, Universidad Nacional de Colombia, Sede Bogotá, Carrera 30, No. 45-03, Bogotá 111321, Colombia; 4 Nature Research Centre, Institute of Ecology, Vilnius, Lithuania; 5 Biology Department, Institute of Genomics and Evolutionary Medicine (iGEM), Temple University, Philadelphia, Pennsylvania 19122-1801, USA

**Keywords:** Erythrocytic non-pigmented parasite, *Garnia*, lizards, *Plasmodium*

## Abstract

Morphological traits from blood stages have been the gold standard for determining haemosporidian parasite species. However, the status of some taxa and the value of such traits in parasites from reptiles remain contentious. The scarce sampling of these species worsens the situation, and several taxa lack molecular data. A survey was performed in the Magdalena Department in Colombia, where 16 species of reptiles were captured. A peculiar haemosporidian parasite was found in the Turnip-tailed gecko *Thecadactylus rapicauda*. This haemosporidian does not show malarial pigment in blood stages under light microscopy; thus, it fits the *Garnia* genus's characters belonging to the *Garniidae*. However, the phylogenetic analyses using a partial sequence of *cytochrome b* and the mitochondrial DNA placed it within the *Plasmodium* clade. Our findings suggest that many putative *Garnia* species belong to the genus *Plasmodium*, like the one reported here. This study either shows that visible malarial pigment in blood stages is not a diagnostic trait of the genus *Plasmodium* or malarial pigment might be present in an undetectable form under a light microscope. In any case, the current taxonomy of haemosporidian parasites in reptiles requires revision. This study highlights the importance of using molecular and morphological traits to address taxonomic questions at the species and genus levels in haemosporidian parasites from reptiles.

## Graphical abstract

**Figure:**
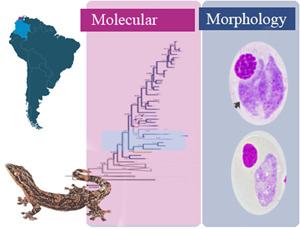

## Introduction

According to their morphological traits, Garnham (1966) classified haemosporidian parasites (Haemosporida, Apicomplexa) into 3 families – Haemoproteidae, Leucocytozoidae and Plasmodiidae. However, Lainson *et al*. (1971) created a fourth family, Garniidae. Those malaria-like parasites belonging to Garniidae were considered similar to *Plasmodium* species by the presence of merogony in blood cells. Still, they were set apart due to the absence of visible malarial pigment granules (haemozoin) in blood stages (meronts and gametocytes), including the stages developing in red blood cells. Family Garniidae contains 3 genera *Garnia*, *Fallisia* and *Progarnia*. *Garnia* species develop only in red blood cells, *Fallisia* spp. develop only in thrombocytes or leucocytes (Lainson *et al*., 1974) and *Progarnia* spp. develop in red blood cells, thrombocytes or leucocytes (Lainson, 1995). The latter parasites were found only in crocodiles. Garniidae parasites have been reported in birds and reptiles (Gabaldon *et al*., 1985; Lainson, 2012). However, the status of such genera has been controversial.

Telford (1973) did not accept the family Garniidae as a valid taxon and considered it a synonymy of Plasmodiidae. Likewise, the genus *Garnia* was also proposed to be a synonymy of *Plasmodium* (Telford, 1973). However, the possibility of a subgenus *Garnia* under the genus *Plasmodium* was kept open for consideration if evidence was provided. This taxonomic proposal was based on an experimental infection using an isolate of *Garnia telfordi*, in which he observed blood stages both containing and not containing visible pigment granules. To validate this taxonomic change, Telford (1973) suggested broadening a definition for the Plasmodiidae, including the parasites that do not contain visible malarial pigment at some stages of development in blood. Others adopted this proposal (Ayala, 1978). However, Garnham and Duggan (1986); Boulard *et al*. (1987); Paperna and Landau (1990*a*); Diniz *et al*. (2000) and Valkiūnas (2005) considered this taxonomic change premature based on limited morphological observations and still considered Garniidae as a family of the Haemosporida.

Nevertheless, recent molecular phylogenies have supported Telford's (1973) opinion regarding Garniidae. It was shown that some Garniidae species likely belong to *Plasmodium* because the parasites lacking hemozoin in blood stages were placed along with *Plasmodium* species in phylogenetic hypotheses constructed with *cytochrome b* (*cytb*) fragments and mitochondrial genomes (Perkins, 2000; Córdoba *et al*., 2021). For example, *Plasmodium ouropretensis* features fit with the characteristics of *Fallisia* parasites due to infection of white blood cells and thrombocytes. Other examples of unpigmented malarial parasites are *Plasmodium leucocytica* and *Plasmodium azurophilum* (Perkins, 2000).

In a recent expedition developed in the Sierra Nevada de Santa Marta in the Caribbean zone of Colombia, a parasite lacking visual malarial pigment was detected infecting a specimen of Turnip-tailed gecko (*Thecadactylus rapicauda*). This paper aimed to characterize this unpigmented haemosporidian parasite.

Only a handful of studies have reported the frequency of *Garnia*-like parasites (Picelli *et al*., 2020). Most of the information about the distribution of these haemosporidians has been obtained using microscopic examination of blood films, while molecular data are available only for 5 species (Perkins, 2000; Córdoba *et al*., 2021). Thus, this study provides molecular evidence that will further our understanding of the diversity and phylogenetic relationships of haemosporidian parasite species without visible malarial pigment in blood stages under light microscopy.

## Materials and methods

### Study area and sample collection

Sampling was performed in 2 localities of the Magdalena Department: the surroundings of ‘Santa Marta’ and ‘El Congo’ biological stations. In total, 26 reptiles belonging to 16 species were captured. Only 1 specimen of Turnip-tailed gecko (*T. rapicauda*) was captured manually at ‘El Congo’ biological Station belonging to ‘Pro-Sierra Nevada de Santa Marta’ Foundation (10.99N, −74.06W; 980 m above sea level). The ‘Sierra Nevada de Santa Marta’ is an isolated mountain range of Colombia located north beside the Caribbean Sea, with an annual precipitation of less than 2000 mm and a mean annual temperature under 20°C (Restrepo *et al*., 2019).

The Turnip-tailed gecko, which most probably corresponds to a species complex (Kronauer *et al*., 2005), has a wide geographical distribution in the New World, being recorded in northern South America: Venezuela, the Guianas, Brazil, both sides of the Andes in Ecuador and Colombia, and the eastern side of Peru and Bolivia; Central America up to Mexico and in the Lesser Antilles (Avila-Pires, 1995). It is a relatively large, primarily arboreal lizard found in primary and secondary forests and sometimes in houses or animal shelters close to patches of trees. It is principally nocturnal in habits and spends the daylight hours under cover of loose bark, hollow trees and other secluded retreats, and it may also be found on the ground (Russell and Bauer, 2002).

### Microscopic examination and parasite morphology

After the specimen was captured, the puncture of the caudal vein was performed to obtain 3 thin blood smears and blood drops were stored in absolute ethanol. Smears were air-dried, fixed with absolute methanol for 5 min and stained with 4% Giemsa for 45 min (Rodríguez and Matta, 2001). Later, they were examined double-blind using an Olympus BX43 microscope (Olympus Corporation, Tokio, Japan). Parasites were photographed with CellSens (Olympus Corporation). Morphometric features studied were those described by Lainson and Naiff (1999) and Valkiūnas (2005). At least 100 images of the parasite were obtained and analysed and ImageJ (Schneider *et al*., 2012) was used to obtain measurements. The parasitaemia was estimated at 1000× magnification, measuring the percentage of parasites where blood cells formed a monolayer (no. of parasites/10 000 erythrocytes) (Staats and Schall, 1996).

### DNA extraction and mitochondrial genome (mtDNA) amplification

DNA was extracted from the whole blood of the only haemosporidian parasite microscopy-positive Turnip-tailed gecko using the QIAamp DNA Micro Kit (Qiagen GmbH, Hilden, Germany). Partial parasite mitochondrial DNA genome (mtDNA, 5884 bp) was obtained using a nested polymerase chain reaction (PCR) protocol with Takara LA Taq™ polymerase (TaKaRa Takara Mirus Bio) following Pacheco *et al*. (2018, 2020). The mtDNA was amplified using the outer oligos forward AE170-5′ GAGGATTCTCTCCACACTTCAATTCGTACTTC 3′ and reverse AE171-5′ CAGGAAAATWATAGACCGAACCTTGGACTC 3′, and the inner oligos forward AE176-5′ TTTCATCCTTAAATCTCGTAAC 3′ and reverse AE136-5′ GACCGAACCTTGGACTCTT 3′. PCR reactions were carried out in 50 *μ*L, and negative (dH_2_O) and positive controls (samples from infected humans) were included. Five *μ*L of the total DNA was used for the primary PCR, and then 1 *μ*L of the PCR product was used for the nested PCR. Amplification conditions for both PCRs were a partial denaturation at 94°C for 1 min and 30 cycles with 30 s at 94°C and 7 min at 67°C, followed by a final extension of 10 min at 72°C. At least 2 independent nested PCR products (50 *μ*L) were excised from the gel (bands of ~6 kb), purified using the QIAquick Gel extraction kit (Qiagen, GmbH, Hilden, Germany) and cloned into the pGEM-T Easy Vector systems (Promega, Madison, USA) following the manufacturer's instructions. Both strands of 3 clones were sequenced using an Applied Biosystems 3730 capillary sequencer. Inconsistencies between the clones were not found, and no mixed infection (2 distinct parasite species) was detected. The mtDNA genome sequence obtained in this study was identified as *Plasmodium* using BLAST (Altschul *et al*., 1997) and submitted to GenBank under accession number ON161138.

### Phylogenetic analyses

Phylogenetic relationships between the lineage found in the Turnip-tailed gecko and other haemosporidian parasites infecting lizards were inferred. Two alignments were constructed using ClustalX v2.0.12 and Muscle as implemented in SeaView v4.3.5 (Gouy *et al*., 2010) with manual editing. The first alignment included 80 partial *cytb* gene sequences (410 bp excluding gaps) belonging to 4 genera (*Leucocytozoon*, *Haemoproteus*, *Haemocystidium* and *Plasmodium*) available from GenBank and the *cytb* sequence extracted from the mtDNA genome obtained in this study. This partial sequence of *cytb* gene is the most commonly sequenced fragment (460–1113 bp out of 1131 bp) that allows broader comparisons between the new sequence obtained from the Turnip-tailed gecko and those from other reptilian *Plasmodium* parasites deposited in the public database GenBank (Benson *et al*., 2012). A second alignment (5242 bp excluding gaps) was done using 58 mtDNA genome sequences of parasites belonging to 4 genera (*Leucocytozoon*, *Haemoproteus*, *Haemocystidium* and *Plasmodium*), including the mtDNA genome reported here (ON161138) and those available in the GenBank (Benson *et al*., 2012). The phylogenetic relationships were inferred using 6 partitions (Pacheco *et al*., 2018). Although the mtDNA genome has more informative sites and yielded a better phylogenetic signal than the small *cytb* fragment (410 *vs* 5242 bp excluding gaps), the second alignment had fewer lineages (*N* = 81 *vs* 58) given the lack of data from those haemosporidian parasites infecting reptiles (*N* = 12).

Then, 2 phylogenetic hypotheses were inferred based on those alignments. Trees were estimated using a Bayesian method implemented in MrBayes v3.2.6 with the default priors (Ronquist and Huelsenbeck, 2003) and a general time-reversible model with gamma-distributed substitution rates and a proportion of invariant sites (GTR + Γ + I). This was the best model that fits the data with the lowest Bayesian information criterion scores, as estimated by MEGA v7.0.14 (Kumar *et al*., 2016). In both analyses, Bayesian support was inferred for the nodes in MrBayes by sampling every 1000 generations from 2 independent chains lasting 2 × 106 Markov Chain Monte Carlo steps. The chains were assumed to have converged once the potential scale reduction factor value was between 1.00 and 1.02, and the average s.d. of the posterior probability was <0.01. Then, 25% of the samples were discarded once convergence was reached as a ‘burn-in’. GenBank accession numbers of all sequences (*cytb* and mtDNA genomes) used in these analyses are shown in the phylogenetic trees. Also, the average evolutionary divergence over all sequence pairs was estimated using both alignments (mtDNA and partial *cytb* gene) and the Kimura 2-parameter model (Kimura, 1980) in MEGA v7.0.14 (Kumar *et al*., 2016).

## Results

**Fig. 1.**
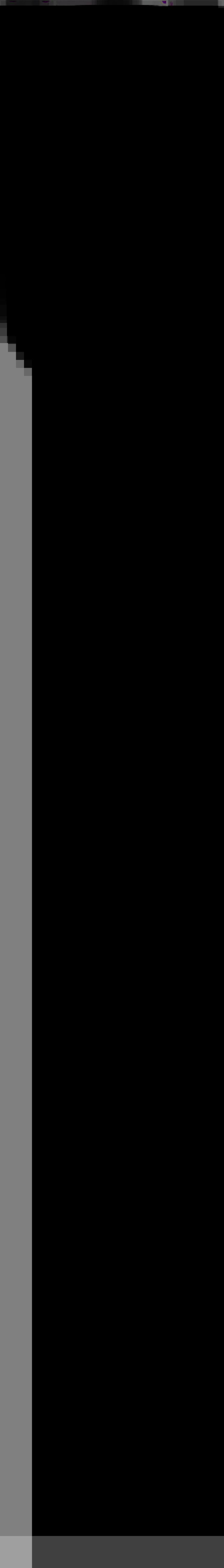
Macrogametocytes (a–e) and microgametocytes (g–h) of a non-pigmented *Plasmodium* TERAP_01 EB256PB were found in *Thecadactylus rapicauda*. Scale bar = 10 *μ*m. Triangle-headed arrow: granules in the cytoplasm. Black triangle: A small space, like a capsule surrounding the parasite. Two-headed arrow: Space between parasite and nucleus of the erythrocyte. Fine black arrow: small vacuoles. Bold black arrow: Nucleus of the parasite. Asterisk: a cytoplasmic space.

A haemosporidian parasite lacking malarial pigment was found in the blood of the Turnip-tailed gecko (Fig. 1). This parasite certainly belonged to Haemosporida based on the evident sex dimorphism observed in gametocytes (see description below). Based on phylogenetic analyses, this parasite was identified as *Plasmodium* sp. lineage TERAP_01. The parasitemia was 0.86%. Because only gametocytes were present (no trophozoites or meronts were observed), we consider prematurely reporting a species' description.

### Description of *Plasmodium* sp. (lineage TERAP_01)

Gametocytes have variable shapes, being predominantly of fusiform with more or less narrowed ends (Fig. 1a–c, f–h, Table 1). It is important to highlight that they possess numerous tiny (dust-like) reddish volutin granules, which are not refractive and readily distinguishable from true malarial pigment (hemozoin). Volutin is often present in gametocytes of haemosporidian parasites (Valkiūnas, 2005). None of the observed gametocytes adhere to the erythrocyte nuclei (Fig. 1). They were predominantly located laterally to the nuclei. The volutin granules and the small vacuoles (0.08–0.89 *μ*m, Table 1) were randomly scattered in the cytoplasm (Fig. 1). A thin band-like space, which appears like the pale-stained cytoplasm of host cells, was often visible around gametocytes (Fig. 1a, g, h).

**Table 1.** Morphometric measurements (in *μ*m) (range followed by mean ± standard deviations in parentheses) of *Plasmodium* MAG026 (EB256PB) found in Turnip-tailed gecko (*Thecadactylus rapicauda*)

Parameter	*Plasmodium* (*Garnia*) sp.	*Garnia karyolytica* (Lainson and Naiff, 1999)
Uninfected erythrocyte	*n* = 10	
Length	21.30 ± 2.19 (18.67–24.04)	
Width	14.05 ± 1.58 (12.10–16.32)	
Area	242.10 ± 48.34 (185.84–307.69)	
Uninfected erythrocyte nucleus		
Length	9.14 ± 1.33 (7.39–12.07)	
Width	5.94 ± 0.76 (5.21–7.17)	
Area	43.84 ± 9.96 (35.91–65.08)	
*Macrogametocytes fusiforms* (Fig. 1a and b)		
Infected erythrocyte	*n* = 7	
Length	21.83 ± 3.67 (16.32–27.20)	
Width	14.95 ± 3.52 (12.97–14.95)	
Area	246.57 ± 37.07 (186.89–281.27)	
Infected erythrocyte nucleus		
Length	9.09 ± 1.79 (7.47–9.99)	
Width	6.02 ± 1.50 (4.18–6.99)	
Area	44.78 ± 18.31 (28.33–54.03)	
Gametocyte	*n* = 11	
Length	27.81 ± 31.73 (13.68–117.86)	
Width	6.52 ± 0.86 (5.12–7.52)	
Area	81.10 ± 13.34 (66.87–97.41)	
Gametocyte nucleus		
Length	3.66 ± 1.79 (1.93–5.70)	
Width	2.91 ± 2.91 (1.63–4.22)	
Area	8.45 ± 2.45 (5.92–12.42)	
Pigment granules	0.21 ± 0.15 (0.10–0.89)	
*Elongate form* (Fig. 1f)		
Infected erythrocyte	*n* = 1	
Length	17.9	
Width	15.10	
Area	209.39	
Infected erythrocyte nucleus		
Length	8.03	
Width	5.59	
Area	36.64	
Gametocyte	*n* = 4	*n* = 50
Length	18.01 ± 1.63 (16.07–20.04)	16.6 (13.3–21.4)
Width	6.87 ± 0.48 (6.29–6.06)	6.3 (4.4–8.1)
Area	106.86 ± 5.37 (100.36–108.65)	–
Gametocyte nucleus		
Length	3.28 ± 0.57 (2.59–3.61)	
Width	3.39 ± 0.79 (0.79–5.80)	
Area	14.18 ± 1.51 (12.17–15.83)	
Pigment granules	0.16 ± 0.06 (0.09–0.28)	
*Round-shaped form* (Fig. 1e)		
Gametocyte	*n* = 2	*n* = 13
Length	11.25 ± 1.18 (10.41–12.08)	9.5 (7.4–11.1)
Width	8.03 ± 0.30 (7.82–8.24)	8.0 (6.6–9.6)
Area	71.80 ± 9.06 (65.39–78.20)	
Gametocyte nucleus		
Length	3.16 ± 0.88 (2.53–3.78)	
Width	3.08 ± 0.37 (2.82–3.34)	
Area	7.29 ± 0.47 (7.60–8.27)	
Pigment granules	0.21 ± 0.07 (0.14–0.37)	
*Microgametocytes*		
Infected erythrocyte	*n* = 2	
Length	20.49 ± 1.05 (19.75–21.23)	
Width	13.79 ± 0.43 (13.48–14.09)	
Area	215.28 ± 9.46 (208.59–221.97)	
Infected erythrocyte nucleus		
Length	8.71 ± 1.53 (7.63–9.79)	
Width	5.61 ± 0.65 (5.15–6.07)	
Area	38.26 ± 7.20 (33.07–43.25)	
Gametocyte	*n* = 10	*n* = 50
Length	16.76 ± 5.40 (6.17–20.57)	15.25 (12.6–18.5)
Width	6.97 ± 1.28 (5.34–9.96)	6.24 (4.4–8.1)
Area	78.69 ± 19.76 (57.32–119.48)	
Gametocyte nucleus		
Length	3.01 ± 3.97 (0.93–13.38)	
Width	2.36 ± 1.85 (0.54–5.93)	
Area	9.44 ± 16.78 (1.00–50.37)	
Volutin pigment granules	0.17 ± 0.10 (0.08–0.76)	

Measurements of *G. karyolytica* found in the same host species were provided for comparison.

Macrogametocytes (Fig. 1a–j) were fusiform (79%) (Fig. 1a and b, Table 1), halteridial (9%) (Fig. 1c, Table 1) and round-shaped form (2%, Fig. 1e, Table 1). Some circumnuclear macrogametocytes (6%), which nearly surround the nuclei of erythrocytes without displacing the nuclei, were seen occasionally (Fig. 1d, Table 1). Multiple infections of 1 host cell with 2 gametocytes were seen (Fig. 1f, Table 1).

Microgametocytes (Fig. 1k and l) have pale staining of the cytoplasm and diffuse nuclei compared to macrogametocytes. The nuclei occupy approximately 1/3 of the parasite cells in the microgametocytes (Fig. 1g and h). The nuclei are pale and poorly distinguishable from the cytoplasm; the reddish nucleole-like structure was conspicuous and visible in all microgametocytes (Fig. 1f–h). Microgametocytes are generally located laterally to nuclei of erythrocytes, and they displace the nuclei laterally (Fig. 1k and l).

### Phylogenetic relationships

**Fig. 2.**
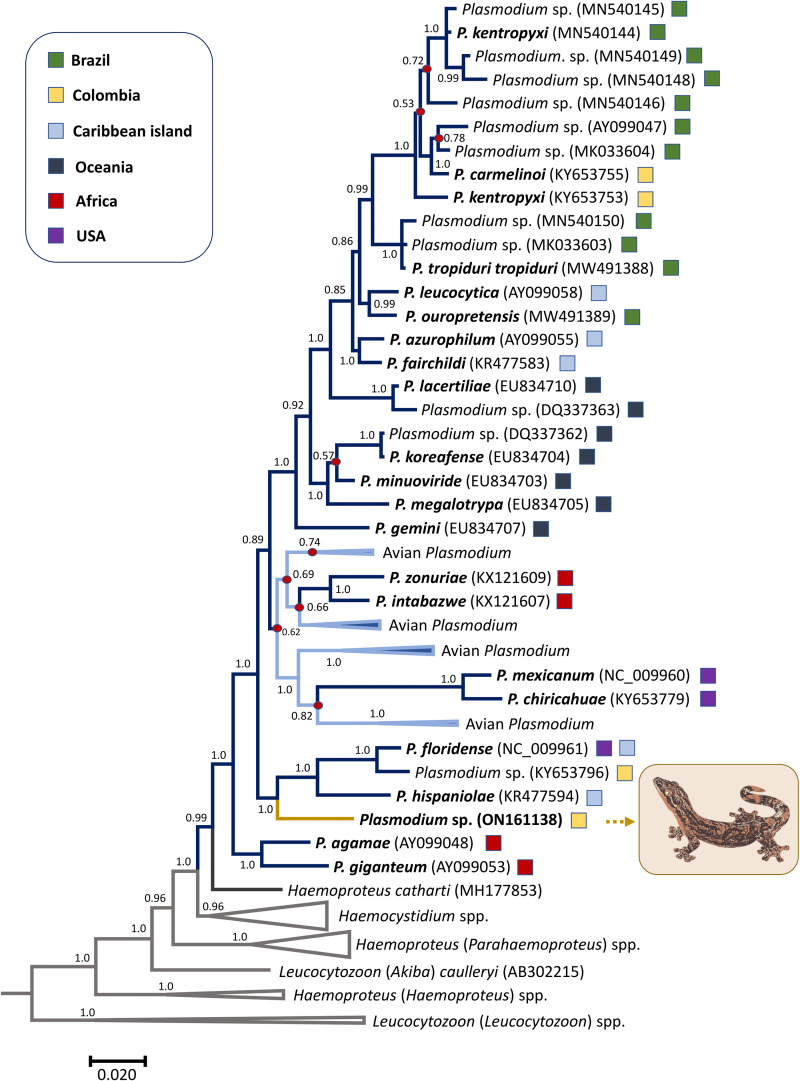
Bayesian phylogenetic hypothesis of reptilian haemosporidian parasites based on partial *cytb* gene (410 bp excluding gaps). The values at the nodes are posterior probabilities, and parasites described as morphospecies are in bold. Branch colours indicate different genera/hosts. Grey branches show the species used as an outgroup. GenBank accession numbers for all parasite sequences used in this analysis are provided in parentheses, and the geographic origins of the sequences are indicated with a coloured square.

**Fig. 3.**
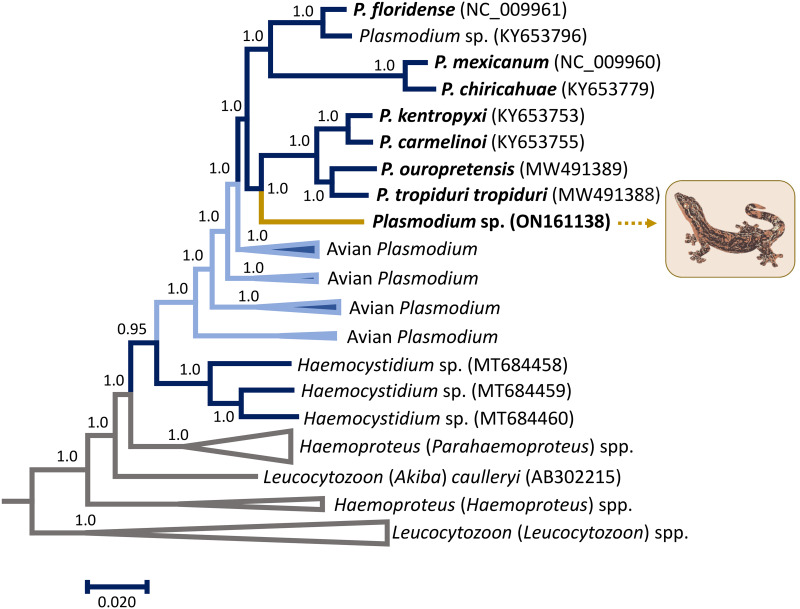
Bayesian phylogenetic hypothesis of reptilian haemosporidian parasites based on mitochondrial genomes (mtDNA, 5242 bp excluding gaps). Parasites' names described also using morphology are given in bold. Branch colours indicate different genera/hosts. Grey branches indicate the species used as an outgroup. GenBank accession numbers for all parasite sequences used in this analysis are provided in parentheses.

Figures 2 and 3 show the phylogenetic relationships between the *Plasmodium* sp. TERAP_01 found in Turnip-tailed gecko and other reptilian parasites with partial *cytb* gene (Fig. 2) and mitochondrial genomes (mtDNA, Fig. 3). The phylogenies, overall, coincide with those previously reported that included other parasites from reptiles (González *et al*., 2019; Pacheco *et al*., 2020; Córdoba *et al*., 2021). Both phylogenetic hypotheses showed that the parasite found in Turnip-tailed gecko shares a common ancestor with *Plasmodium* species found in lizards. In the phylogeny using partial *cytb* sequences, the parasite reported in this study shares a common ancestor with *Plasmodium floridense*, *Plasmodium hispaniolae* and *Plasmodium* (*Lacertamoeba*) sp., which are species that produce hemozoin, and all are from the Caribbean region (Fig. 2). Nevertheless, it is distantly related to other non-pigmented species like *Plasmodium azurophylum*, *P. leucocytica* and *P. ouropretensis*, with genetic distances of 0.059 ± 0.012, 0.062 ± 0.013 and 0.062 ± 0.012, respectively (Fig. 2, Table 2 and Supplementary Table S1).

**Table 2.** Pairwise genetic distance among *Plasmodium* species (lineages MAG026, EB256PB) found in Turnip-tailed gecko (*T. rapicauda*) and other reptilian *Plasmodium* spp. using partial *cytb* gene sequences

		Pairwise genetic distance (standard error)						
	Reptilian haemosporidian species	1	2	3	4	5	6	7
1	***Plasmodium* sp. MAG026 (EB256PB)**		0.008	0.009	0.009	**0.008**	**0.009**	**0.009**
2	*Plasmodium hispaniolae* (KR477594)	0.069		0.008	0.007	0.008	0.009	0.010
3	*Plasmodium* (*Lacertamoeba*) sp. (KY653796)	0.077	0.063		0.004	0.009	0.009	0.009
4	*Plasmodium floridense* (NC_009961)	0.075	0.061	0.019		0.009	0.009	0.009
5	***Plasmodium azurophilum* (AY099055)**	**0**.**071**	0.070	0.085	0.076		0.004	0.005
6	***Plasmodium ouropretensis* (MW491389)**	**0**.**076**	0.078	0.089	0.082	0.020		0.004
7	***Plasmodium leucocytica* (AY099058)**	**0**.**076**	0.083	0.089	0.080	0.022	0.018	

Genetic divergence was estimated in MEGA 7.0.18 and the standard error estimate(s) are shown above the diagonal. Given that not all partial *cytb* gene sequences have the same length, for this analysis there were a total of 1045 positions in the final dataset excluding gaps. Genetic divergence between parasites that do not produce haemozoin pigment are show in bold. See Fig. 2 for reference.

It is worth noticing that non-pigmented species do not form a monophyletic group, which is consistent with what has been found recently by Córdoba *et al*. (2021) using similar approaches. However, in the case of the phylogenetic relationships estimated using mtDNA genomes, the *Plasmodium* sp. TERAP_01 found in the Turnip-tailed gecko appears to share a common ancestor with *Plasmodium kentropyxi*, *Plasmodium carmelinoi*, *P. ouropretensis* and *Plasmodium tropiduri tropiduri*. Although the mtDNA genome yielded a better phylogenetic signal than the small *cytb* fragment (Fig. 2 *vs* 3), this result is inconclusive given the lack of molecular data for other reptilian haemosporidian taxa. However, the genetic distance between *Plasmodium* sp. TERAP_01 and the only unpigmented parasite *P. ouropretensis*, for which molecular data of mtDNA genome are available, is 0.063 ± 0.03 (Table 3), similar to the genetic distance estimated with the partial sequence of *cytb* (Table 2).

**Table 3.** Pairwise genetic distance among *Plasmodium* species (MAG026, EB256PB) found in Turnip-tailed gecko (*T. rapicauda*) and other reptilian *Plasmodium* spp. with mtDNA genomes available

		Pairwise genetic distance (standard error)								
	Reptilian haemosporidian species	1	2	3	4	5	6	7	8	9
1	***Plasmodium* MAG026 (EB256PB)**		0.003	**0.003**	0.003	0.003	0.004	0.004	0.004	0.003
2	*Plasmodium tropiduri tropiduri* (MW491388)	0.061		0.002	0.002	0.002	0.003	0.003	0.003	0.003
3	***P. ouropretensis* (MW491389)**	**0**.**063**	0.025		0.002	0.002	0.004	0.004	0.003	0.003
4	*Plasmodium carmelinoi* (KY653755)	0.068	0.032	0.035		0.002	0.004	0.004	0.003	0.003
5	*Plasmodium kentropyxi* (KY653753)	0.068	0.031	0.035	0.015		0.004	0.004	0.003	0.003
6	*Plasmodium mexicanum* (NC_009960)	0.084	0.083	0.081	0.083	0.084		0.002	0.004	0.004
7	*Plasmodium chiricahuae* (KY653779)	0.088	0.087	0.085	0.087	0.088	0.017		0.004	0.004
8	*P. floridense* (NC_009961)	0.063	0.060	0.061	0.062	0.062	0.077	0.079		0.002
9	*Plasmodium* (*Lacertamoeba*) sp. (KY653796)	0.062	0.060	0.063	0.063	0.064	0.076	0.080	0.016	

Genetic divergence was estimated in MEGA 7.0.18 and the Standard error estimate(s) are shown above the diagonal. There were a total of 5426 positions in the final dataset excluding gaps. Genetic divergence between parasites that do not produce haemozoin pigment are show in bold. See Fig. 3 for reference.

## Discussion

Gametocytes of the parasite reported here do not possess visible hemozoin pigment granules. This species infects mature red blood cells and is similar to *Garnia karyolytica* described in the same host species in Brazil (Table 1) (Lainson and Naiff, 1999; Picelli *et al*., 2020). However, the parasite found in Colombia can be readily distinguished because it does not induce lysis of the host cell's nuclei.

This parasite is likely a new species for this host. Three *Plasmodium* species have been reported in the Turnip-tailed gecko in Panama, Brazil and Venezuela. First, *Plasmodium aurulentum*, whose blood stages share features of *P. tropiduri* and *Plasmodium morulum*, develops exo-erythrocytic merogony in thrombocytes and lymphocytes (Telford, 1971). Second, an unidentified *Plasmodium* species (Telford, 1978), and third, *G. karyolytica* (Lainson and Naiff, 1999). The latter 2 haemosporidian have unpigmented blood stages.

It is worth noting that Lainson and Naiff (1999) found only gametocytes in an infected individual of *T. rapicauda* when it was sampled. Two months later, while the infected lizard was maintained in captivity, the other parasite stages, i.e. trophozoites and meronts, were detected. The predominance of gametocytes in peripheral blood was observed in *Plasmodium (Billbraya) australis* in the Australian gecko *Phyllodactylus marmoratus*. Indeed, an occasional and short-lasting erythrocytic merogony was reported (Paperna and Landau, 1990*b*). Whether this is the case with the parasite from Colombia reported here cannot be determined.

The observed malarial pigment is considered a diagnostic trait in *Plasmodium*. The digestion of haemoglobin by *Plasmodium* parasites results in the release of haem, which is toxic for haemosporidians (Egan, 2008). The haem is converted into hemozoin or malarial pigment in developing parasites. However, recent studies have demonstrated that *Plasmodium berghei* can develop even when different genes associated with hemozoin production are disrupted (Lin *et al*., 2015). As a result, the mutant strains do not produce hemozoin, and then undigested haemoglobin remains in vesicles, which confers the parasites’ drug resistance (Lin *et al*., 2015). A study using electron microscopy carried out using *Garnia gonadati* showed that malarial pigment was not detected (Diniz *et al*., 2000), and this parasite lacks a vacuolar system of digestion (Boulard *et al*., 1987). That may indicate an alternative pathway for detoxification in these parasites that is worth studying.

The absence of visible malarial pigment granules has been described during parasitemia in some reptilian *Plasmodium* species, for example, *Plasmodium balli*, *Plasmodium gonatodi* and *P. morulum* (Telford, 1974) as well as *Plasmodium scorzai* and *Plasmodium lainsoni* (Telford, 1988). Unfortunately, there is no molecular information on these parasites. Nevertheless, *Plasmodium* spp. have a markedly different spectrum of malarial pigment morphology (Telford, 2009); e.g. in *P. azurophilum*, pigment granules were seen only in 0.2% of gametocytes (Telford, 1975; Perkins, 2000). Thus visible malarial pigment seems to exhibit phenotypic plasticity in *Plasmodium* from reptiles, questioning its utility to separate taxa, at least among haemosporidian in reptiles (Telford, 1973).

Consistent with this observation (Telford, 1973), the phylogenetic analyses using *cytb* and mitochondrial DNA indicated that the lineage found here shares its most recent common ancestor with other species in the genus *Plasmodium*. Further, this parasite does not form a monophyletic group with other parasites without malarial pigments, such as *P. leucocytica*, *P. azurophilum* and *P. ouropretensis* (Fig. 2).

Based on the results presented here, *Plasmodium* sp. TERAP_01 and other unpigmented parasites may have originated independently from evolutionarily distinct lineages (Figs 2 and 3). This is consistent with the observation that visible malarial pigment is a variable character in reptile haemosporidia (Telford, 1973) and the fact that unpigmented parasites are not a monophyletic group (Perkins, 2000; Galen *et al*., 2018; Córdoba *et al*., 2021). Perhaps, visible pigment production could have been gained or lost throughout the evolutionary history of these parasites, as has been proposed by Galen *et al*. (2018).

There is a discussion regarding the presence or absence of visible malarial pigment, a debate that can be separated into 2 non-mutually exclusive issues: whether there is truly no malarial pigment in some parasites and how valuable this trait is, presence or absence of visible malarial pigment, as a diagnostic tool for a taxon such as Garniidae. Regarding the first issue, part of the problem is that these reptile parasites' biology and life cycles remain insufficiently studied. Hemozoin may appear only in specific stages of the life cycle. Long-lasting experimental observations are needed to answer this question, but such studies remain rare. Perhaps, a more sensitive technique, such as flow cytometry or histochemistry, could detect malarial pigment in some haemosporidian reptile parasites even below the detection by microscopy (Rebelo *et al*., 2013; Orbán *et al*., 2014).

We now return to the question posed above i.e., is visible malarial pigment a valuable trait for diagnosing the genus *Plasmodium* or creating a taxon such as Garniidae? (Telford, 1973). Indeed, it is difficult to rule out that some parasites classified as *Garnia* spp. have few discrete dust-like hemozoin granules, which are difficult to detect by light microscopy, the tool used in the classical taxonomy of Haemosporida. As indicated earlier, *Plasmodium* spp. have a broad spectrum of malarial pigment morphology (Telford, 1975, 2009; Perkins, 2000; Noland, *et al*., 2003). Thus, the evidence suggests that visible malarial pigment is not a valuable trait for separating taxa (Telford, 1973).

Overall, the taxonomic characters currently used to define the *Plasmodium* genus are not found in all related species of reptile parasites (Figs 2 and 3). Considering that it has been long proposed that *Plasmodium* is a paraphyletic group (Escalante *et al*., 1998; Galen *et al*., 2018; Pacheco *et al*., 2018), the reptile parasites seem to add evidence to this pattern.

Recent studies have suggested different solutions to deal with this taxonomic issue, at least in parasites from reptiles. One of which is to broaden the definition of *Plasmodium*, which in the case of reptiles should include morphological features proposed for *Garnia*, *Fallisia* and *Progarnia* (Telford, 1973; Ayala, 1978; Galen *et al*., 2018). In other words, the definition of the genus *Plasmodium* should be broadened to include parasites with and without visible hemozoin under the light microscope and should also consider parasites that are capable of infecting various red blood cells, leucocytes and thrombocytes as part of the genus.

Due to the incomplete knowledge of the biology of putative Garniidae species, it would be logical not to make changes in the taxonomy until information on the biology and molecular systematics of more putatively garniid species is available. Although molecular phylogenies that include parasites from reptiles are still limited (Perkins, 2000; Galen *et al*., 2018; Córdoba *et al*., 2021, and this study), they seem to indicate that Garniidae may not be a valid taxon, as previously proposed (Telford, 1973).

## Conclusion

This study provides a molecular and morphological characterization of the unpigmented parasite *Plasmodium* sp. (lineage TERAP_01) that exhibit *Garnia*-like traits but shares a common ancestor with *Plasmodium* species found in reptiles. Haemosporidian parasites in reptiles remain poorly investigated concerning life cycles and biology, which is particularly true for *Garnia* species. It will be interesting to apply targeting sensitive techniques to detect hemozoin in the blood stages of haemosporidian parasites. Thus far, the molecular evidence seems to question the validity of Garniidae as a family. However, additional studies are required before revising the taxonomy of Haemosporida. In-depth taxonomic sampling and experimental research is necessary to understand better the evolutionary relationships of *Plasmodium* spp. and other haemosporidians, which are remarkably diverse in reptiles.

## Data Availability

The sequence obtained in this study was submitted to GenBank under accession number ON161138. All sequences used in the analyses performed here are available at GenBank.

## Supplementary material

For supplementary material accompanying this paper visit https://doi.org/10.1017/S0031182022001421. click here to view supplementary material
