# Supplementary material for: Morphometric and molecular characterization of an unpigmented haemosporidian parasite in the Neotropical turnip-tailed gecko (Thecadactylus rapicauda)
Source: Parasitology. 2022 Oct 21;150(3):221–9. doi: 10.1017/S0031182022001421 (PMC10090589; doi:10.1017/S0031182022001421)
Supplement: Supplementary file 1 [file S0031182022001421sup001.pdf]

Description and molecular characterization of an unpigmented haemosporidian parasite in the Neotropical Turnip-tailed gecko (*Thecadactylus rapicauda*)

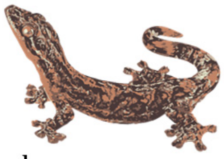

Nubia E. Matta, Leydy P. González, Mario Vargas-Ramírez, Gediminas Valkiūnas, Ananías A. Escalante, M. Andreína Pacheco.

Supplementary Material

Table S1. Estimates of evolutionary divergence among all reptilian *Plasmodium* species included in the phylogenetic tree (Fig. 2). Genetic distances were estimated in MEGA 7.0.18 using partial parasite *cytb* gene (410 bp excluding gaps) sequences. Genetic divergence between unpigmented parasites are shown in bold and Standard Error estimate(s) are shown above the diagonal.

		Pairwise genetic distance (Standard Error)																						
	**Reptilian haemosporidian species**	**1**	**2**	**3**	**4**	**5**	**6**	**7**	**8**	**9**	**10**	**11**	**12**	**13**	**14**	**15**	**16**	**17**	**18**	**19**	**20**	**21**	**22**	**23**
**1**	*P. giganteum* (AY099053)		0.009	0.012	0.014	0.013	0.013	0.016	0.018	0.012	0.010	0.011	0.013	0.010	0.010	0.011	0.010	0.011	0.011	0.012	0.013	0.012	0.011	0.013
**2**	*P. agamae* (AY099048)	0.034		0.014	0.016	0.015	0.014	0.017	0.018	0.014	0.012	0.012	0.014	0.013	0.013	0.014	0.012	0.014	0.013	0.015	0.013	0.014	0.014	0.014
**3**	***Plasmodium* sp. TERAP_01 (ON161138)**	0.057	0.073		0.013	0.012	0.011	0.016	0.018	0.012	0.012	0.014	0.013	0.011	0.012	0.012	0.010	**0.012**	**0.012**	**0.013**	0.012	0.014	0.013	0.013
**4**	*P. hispaniolae* (KR477594)	0.075	0.086	0.062		0.012	0.011	0.018	0.019	0.015	0.015	0.013	0.013	0.013	0.014	0.015	0.013	0.012	0.013	0.014	0.012	0.016	0.015	0.015
**5**	*Plasmodium* (*Lacertamoeba*) sp. (KY653796)	0.075	0.092	0.059	0.054		0.007	0.018	0.019	0.013	0.014	0.014	0.014	0.015	0.015	0.015	0.014	0.014	0.015	0.015	0.013	0.014	0.014	0.014
**6**	*P. floridense* (NC_009961)	0.067	0.081	0.052	0.052	0.021		0.017	0.018	0.014	0.014	0.014	0.014	0.014	0.014	0.014	0.013	0.014	0.014	0.015	0.014	0.013	0.013	0.013
**7**	*P. mexicanum* (NC_009960)	0.106	0.114	0.106	0.120	0.120	0.108		0.006	0.018	0.018	0.016	0.018	0.016	0.017	0.017	0.014	0.015	0.016	0.016	0.017	0.017	0.016	0.018
**8**	*P. chiricahuae* (KY653779)	0.114	0.117	0.114	0.129	0.135	0.123	0.017		0.019	0.019	0.017	0.019	0.017	0.018	0.018	0.015	0.016	0.017	0.017	0.018	0.019	0.017	0.020
**9**	*P. intabazwe* (KX121607)	0.052	0.073	0.052	0.081	0.073	0.070	0.120	0.126		0.009	0.014	0.014	0.013	0.012	0.013	0.011	0.012	0.013	0.013	0.012	0.013	0.013	0.014
**10**	*P. zonuriae* (KX121609)	0.044	0.059	0.057	0.086	0.081	0.073	0.120	0.126	0.036		0.014	0.014	0.013	0.012	0.013	0.011	0.013	0.013	0.014	0.012	0.012	0.012	0.013
**11**	*P. gemini* (EU834707)	0.046	0.062	0.070	0.070	0.075	0.078	0.100	0.103	0.065	0.073		0.013	0.012	0.012	0.012	0.012	0.011	0.012	0.012	0.012	0.013	0.012	0.013
**12**	*P. megalotrypa* (EU834705)	0.067	0.078	0.065	0.070	0.081	0.078	0.114	0.123	0.075	0.083	0.065		0.010	0.011	0.012	0.011	0.012	0.012	0.012	0.013	0.015	0.013	0.014
**13**	*P. minuoviride* (EU834703)	0.049	0.065	0.054	0.067	0.086	0.073	0.100	0.103	0.062	0.065	0.054	0.041		0.007	0.010	0.009	0.010	0.010	0.010	0.011	0.014	0.012	0.013
**14**	*P. koreafense* (EU834704)	0.041	0.067	0.062	0.075	0.083	0.075	0.117	0.126	0.059	0.062	0.057	0.054	0.024		0.012	0.009	0.011	0.011	0.012	0.012	0.013	0.012	0.013
**15**	*P. lacertiliae* (EU834710)	0.052	0.070	0.057	0.075	0.083	0.075	0.106	0.111	0.065	0.062	0.062	0.059	0.041	0.052		0.008	0.010	0.010	0.010	0.010	0.013	0.012	0.012
**16**	*P. fairchildi* (KR477583)	0.044	0.062	0.049	0.062	0.075	0.067	0.089	0.097	0.046	0.049	0.054	0.052	0.034	0.039	0.031		0.007	0.007	0.008	0.008	0.011	0.010	0.011
**17**	***P. azurophilum* (AY099055)**	0.052	0.070	**0.059**	0.059	0.081	0.078	0.097	0.106	0.057	0.065	0.052	0.054	0.044	0.052	0.041	0.019		0.007	0.009	0.008	0.013	0.012	0.013
**18**	***P. ouropretensis* (MW491389)**	0.052	0.065	**0.062**	0.070	0.086	0.078	0.106	0.108	0.059	0.062	0.052	0.054	0.039	0.049	0.041	0.024	0.024		0.007	0.008	0.012	0.011	0.012
**19**	***P. leucocytica* (AY099058)**	0.059	0.081	**0.062**	0.075	0.086	0.078	0.103	0.111	0.062	0.067	0.057	0.059	0.041	0.057	0.044	0.021	0.029	0.021		0.009	0.013	0.012	0.012
**20**	*P. tropiduri tropiduri* (MW491388)	0.062	0.070	0.062	0.062	0.073	0.075	0.108	0.117	0.059	0.062	0.059	0.065	0.049	0.054	0.046	0.029	0.029	0.029	0.031		0.011	0.012	0.012
**21**	*P. kentropyxi* (KY653753)	0.062	0.078	0.078	0.094	0.083	0.078	0.123	0.132	0.067	0.067	0.065	0.086	0.073	0.067	0.070	0.052	0.065	0.062	0.059	0.052		0.007	0.008
**22**	*P. kentropyxi* (MN540144)	0.054	0.070	0.073	0.089	0.081	0.070	0.106	0.120	0.067	0.065	0.062	0.070	0.062	0.062	0.054	0.041	0.057	0.057	0.054	0.057	0.021		0.008
**23**	*P. carmelinoi* (KY653755)	0.073	0.078	0.078	0.089	0.083	0.078	0.129	0.138	0.078	0.078	0.070	0.081	0.073	0.073	0.065	0.057	0.070	0.067	0.065	0.062	0.029	0.026	
